# Multi-Disciplinary Design and Implementation of a Mass Vaccination Clinic Mobile Application to Support Decision-Making

**DOI:** 10.1109/JTEHM.2022.3224740

**Published:** 2022-11-24

**Authors:** Ryan Tennant, Moses Tetui, Kelly Grindrod, Catherine M Burns

**Affiliations:** Department of Systems Design EngineeringUniversity of Waterloo 8430 Waterloo ON N2L 3G1 Canada; Department of Epidemiology and Global HealthUmeå University 901 87 Umeå Sweden; School of PharmacyUniversity of Waterloo 8430 Waterloo ON N2G 1C5 Canada

**Keywords:** Decision support systems, human factors, mass vaccination clinics, mobile applications, usability testing

## Abstract

Mass vaccination clinics are complex systems that combine professionals who do not typically work together. Coordinating vaccine preparation and patient intake is critically important to maintain patient flow equilibrium, requiring continuous communication and shared decision-making to reduce vaccine waste. Objectives: (1) To develop a mobile application (app) that can address the information needs of vaccination clinic stakeholders for end-of-day doses decision-making in mass immunization settings; and (2) to understand usability and clinical implementation among multi-disciplinary users. Methods: Contextual inquiry guided 71.5 hours of observations to inform design characteristics. Rapid iterative testing and evaluation were performed to validate and improve the design. Usability and integration were evaluated through observations, interviews, and the system usability scale. Results: Designing the app required consolidating contextual factors to support information and workload needs. Twenty-four participants used the app at four clinics who reported its effectiveness in reducing stress and improving communication efficiency and satisfaction. They also discussed positive workflow changes and design recommendations to improve its usefulness. The average system usability score was 87 (n = 22). Discussion: There is significant potential for mobile apps to improve workflow efficiencies for information sharing and decision-making in vaccination clinics when designed for established cultures and usability, thereby providing frontline workers with greater time to focus on patient care and immunization needs. However, designing and implementing digital systems for dynamic settings is challenging when healthcare teams constantly adapt to evolving complexities. System-level barriers to adoption require further investigation. Future research should explore the implementation of the app within global contexts.

## I. Introduction

Vaccination clinics have been a successful and essential strategy for bringing the COVID-19 vaccines to the public quickly and effectively [1]. Yet, they can also be stressful and chaotic for frontline workers [2], which may negatively impact the patient experience. Successfully managing a mass vaccination clinic involves coordinating and communicating information among stakeholders such as pharmacists, nurses, physicians, students, non-clinical staff, security staff, and volunteers in an environment where they may not usually work together so closely [3]. These environments are also typically not designed for mass immunization or supporting the physical and cognitive demands for effectively, efficiently, and safely vaccinating large communities.

**FIGURE 1.**
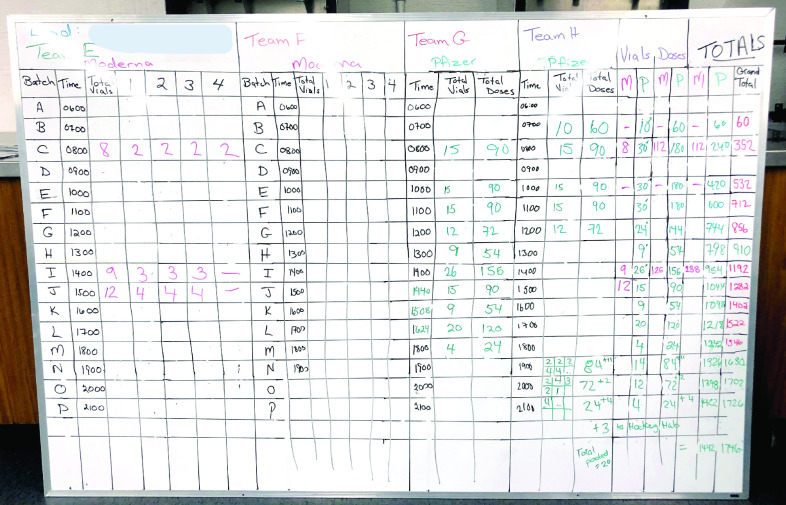
Mass vaccination clinic doses tracking whiteboard for a single-day vaccination event in the Region of Waterloo, providing two brands of the COVID-19 vaccine. Personal identifying information is redacted.

One critical task for coordinating a mass vaccine clinic involves managing open vial waste and end-of-day doses [4], which requires aggregating patient intake with vaccine preparation information while continuously determining the number of doses needed. This task becomes particularly challenging with variations that occur in patient intake, the number of doses available per vial for a vaccine brand, and the desire to minimize waste. While most of the work is completed manually (Figure 1) contributing to a high workload especially if errors occur in tracking or calculations, this demanding task reduces time for frontline workers to spend on maintaining patient safety and immunization needs and supporting medical emergencies.

There is potential for supporting the cognitive workload associated with handling information that is constantly changing in dynamic healthcare settings through the design and implementation of electronic systems. A human factors approach, focusing on users within their work domain combined with systems science, has successfully supported technology design and development in other healthcare settings [5], [6], [7]. However, to the best of the authors’ knowledge, there are no implemented electronic systems augmenting decision-making aspects of vaccination clinics.

### A. Background

Ensuring high-quality electronic data within mass vaccination clinics is fundamental to supporting workflow and patient care [8]. In Canada, there is significant potential to support mass vaccination clinic workflow through electronic system development [9], [10]. A study in the context of the H1N1 immunization campaign from 2009 across nine provinces and 300 participants demonstrated a willingness to integrate novel technologies that support data collection on vaccination records in clinics, despite potential issues with usability [9]. Barcode scanning systems were also perceived to improve patient safety and accuracy of records because the data was digitalized [10].

Despite the potential benefits of implementing nationwide electronic systems in healthcare Canada does not have a national electronic vaccination system, likely due to politically driven relationships among healthcare providers and decentralized administration among the provinces [11], [12]. Similar barriers were likely the reasons why there is not a nationwide electronic system to support mass vaccination clinics during the COVID-19 pandemic. As a result, provinces and regions acquired their own systems.

In the Region of Waterloo, Ontario, Canada, vaccination clinics required the rapid development of electronic systems for managing appointment bookings and recording vaccine administration. Private companies developed these systems quickly to meet vaccination demands. However, their design and implementation do not support real-time calculations to manage patient flow, vaccine preparation rates, and reduce vaccine waste. These factors have been challenging for mass vaccination clinics during the COVID-19 vaccination rollout [3]. Thus, to account for the missing information and communication needs, clinic staff created their own tools and artifacts.

Improving mass vaccination clinic workflow through modelling and technology development is an emerging area of research interest [4], [13], [14]. It has never been more critically important to inform the design and development of healthcare technologies by understanding the specific communication, coordination and information needs of multi-disciplinary stakeholders to design and implement technologies that will solve systemic inefficiencies.

To the best of the authors’ knowledge, this research study is the first to report on the rapid participatory, multi-disciplinary design, development, and evaluation of a novel mobile application (app) for COVID-19 mass vaccination clinics. Our study highlights the overarching importance of a multi-disciplinary human factors approach to design, despite requiring rapid development of software systems in dynamic public health delivery settings.

### B. Objectives of the Study

This study involved designing, developing, and evaluating a mass vaccination clinic mobile app in the Region of Waterloo. The objectives were two-fold:

1. To develop a mobile app that can address the information needs of multi-disciplinary vaccination clinic staff for end-of-day doses decision-making.
2. To understand the usability and integration of the app within mass vaccination clinic settings.

## II. Methods and Procedures

This study was one component of a larger research project on mass vaccination clinics, which was initially approved and received ethics clearance from a University of Waterloo Research Ethics Committee (May 27^th^, 2021: #43288), the Region of Waterloo Public Health and Emergency Services and the Tri-Hospital Research Ethics Review Board (Jun 3^rd^, 2021: #2021-0735). Eligible participants included staff at clinics who were tracking patient numbers or preparing the vaccine. Data was collected at mass vaccination clinics in the Region of Waterloo.

### A. Clinic Observations and Contextual Inquiry

Observations were conducted to identify challenges and opportunities for improvements. RT and MT made detailed notes about everything they observed to identify design requirements. Notetaking was guided by the contextual inquiry and contextual design frameworks [15]. At the end of each day, they consolidated and reflected on their observations. On a weekly basis a reflective meeting that involved KG and CB, both highly experienced in vaccine clinic and system design processes respectively, was undertaken to conceptualize the observations. Consequently, the data were summarized by developing contextual design models [15], which identified challenges, stress points, and clinic improvement opportunities. The detailed results of the contextual inquiry and design models that were used to inform the engineering design were previously published [16].

At each clinic, the staff responsible for decisions regarding end-of-day doses were closely followed. A master-apprentice approach was taken during the observations, where RT and MT asked clarification questions based on the activities the clinic staff were performing. Data were primarily collected on activities involving calculations, communicating with others, finding information, and addressing challenges as they arose. Questions were asked to clinic staff to clarify observation data without disrupting their workflow. Clinic staff also participated in semi-structured interviews during breaks in the clinic to clarify roles, responsibilities, strategies.

### B. Application Design & Development

Initial design requirements were identified from the consolidated observation data across clinics. Based on prior expertise, the desire to control app deployment to consenting participants, and the potential risk of excessive traffic on a web-app, we built a native mobile app. The app was developed by RT using React-Native and a Canadian Google Cloud Firestore database and deployed on iOS and Android using Expo Go. Following guidance from the University of Waterloo’s Information Systems & Technology team, server-side and in-app rules for user role types were implemented to mitigate potential input errors by unauthorized users and to ensure authenticated access to clinic profiles. The security measures were evaluated manually with *Test User* accounts.

Building a functional prototype was necessary at this stage in the mass vaccination program to encourage meaningful engagement in the iterative design process and support future use and adoption. Rapidly developing and providing clinical staff with the data-driven app supported clinic staff to quickly understand the potential for such a tool to support real-time challenges.

### C. Rapid Iterative Testing and Evaluation

The researchers employed the Rapid Iterative Testing and Evaluation (RITE) process to identify issues quickly and collaboratively with system usability. RITE is emerging as a popular approach for game designers, software developers and health technology researchers for its success in quickly revealing usability issues that might otherwise be difficult to identify [17], [18], [19], [20], [21]. Unlike a standard usability test where all participants are tested before making design changes, updates to the user interface are made once the issue is identified and understood, even after one participant [22]. Further testing is completed to determine if the problem has been appropriately resolved and the process is continued until no additional issues emerge [22].

The RITE method is used with working software. Therefore, this requires the ability to identify usability issues from researchers who have domain knowledge of the working environment and the ability to address software issues [18]. One benefit of this methodology is that it can identify more concerns within a short amount of time [22]. A participatory iteration process can also enhance user engagement as participants can see their problems or feedback directly implemented as the design is iterated upon [22], [23]. In the context of healthcare, rapid iterative testing is fundamentally important to understand how implementing technologies may influence workflow [24].

Participants of the three clinics were recruited via email to download the app onto a mobile device at their clinic. Before participating, the researchers provided a verbal or video orientation. Participants were asked to perform the tasks listed in Table 1 in a *Test Clinic* profile which was video recorded. They were asked only to complete the tasks relevant to their role at the clinic. The results were qualitatively analyzed to identify challenges navigating the interface. Participants verbally reported their experiences after completing the tasks; their feedback was audio recorded. As usability issues were identified from the videos and feedback, they were iteratively addressed through software updates for subsequent testing until no new issues were identified.

**TABLE 1:** Usability Measurement Tasks

Task #	Task Prompts
1	There are 809 total appointments for today.^a^
2	There are 140 vials stored in the fridge for today.^b^
3	At the clinic, the vaccine being used today is Pfizer.^c^
4	There were 24 walk-ins since the start of your shift.^a^
5	50 vials have been opened so far.^b^
6	$A$ total of 18 residual doses from pooling has been made.^b^
7	The total number of appointments changed from 809 to 808 for today.^a^
8	One dose was wasted due to suspected contamination.^b^
9	There were 18 no-shows since the start of your shift.^a^
10	Interpret the values on the dashboard to the researcher.^c^

^a^ Expected tasks for the Clinic Lead;

^b^ Expected tasks for the Vaccine Lead;

^c^ Expected tasks for the Clinic Lead and Vaccine Lead.

### D. Implementation Testing

This study used multiple evaluation methods for usability and implementation. After completing the usability testing (Table 1) participants used the app during at least one of their shifts while the researchers observed clinic workflow and how the app was incorporated. They were also encouraged to take notes and contact the researchers with feedback, issues, or questions to enhance the RITE process.

After using the app, participants were asked to partake in an end-of-study interview about their experiences working at the clinic with the app. The interviews were analyzed thematically to identify how users felt the app impacted workflow coordination, and to identify any remaining usability issues. The interviews were conducted either at the clinic or remotely via Microsoft Teams. After the interview, the participants completed the System Usability Scale (SUS) questionnaire, quantitatively assessing the app’s subjective usability [25].

## III. Results

### A. Consolidation for Engineering Design

Observations were conducted at six clinics: the University of Waterloo’s School of Pharmacy (Clinic #1), a large (7,900 to 13,900 m^2^) vacant commercial warehouse (Clinic #2), and the first floor of a medical center (Clinic #3). A two-day mobile clinic in a high school (Clinic #4), a weekend-run clinic in the region’s public health building (Clinic #5) and a temporary two-day mass vaccination clinic at a conference center (Clinic #6) were also included. In total, 71.5 hours were spent observing vaccination clinics. Observations from Clinic #1 and #2 initially informed the wireframes and high-fidelity prototype.

The observations identified several engineering design requirements, constraints, and functions for a collaborative electronic system to support workflow coordination, including potential means (Table 2). A mobile app solution was chosen based on the mobility of clinic staff and the existing use of tablet devices at the clinics. Our approach to building the app focused on meeting requirements and constraints that would best support the characteristics involved with system usability as defined by CSA ISO 9241-11:2018 [26], cognitive workloads for reducing vaccine waste, clinic communication, and operating a safe patient environment.

**TABLE 2:** Informing engineering design from contextual inquiry

	Design Characteristic	R	C	F	M
Effectiveness	1. Provide awareness of clinic state.	✓			
	2. Support cognitive workload.	✓			
	3. Automate dose calculations.			✓	
	4. Display remaining vials/doses.				✓
	5. Store data for all users to view.			✓	
	6. Connect to cloud server.				✓
Efficiency	7. Provide transparency of inputs.			✓	
	8. Aggregate data on one screen.				✓
	9. Displays history of data inputs.				✓
	10. Easy to navigate data inputs.	✓			
	11. Easy to update and edit data.	✓			
	12. Minimal time burden to use.	✓			
Satisfaction	13. Secure data from misuse.		✓		
	14. Control user-clinic access.			✓	
	15. Admin controlled accounts.				✓
	16. Use anywhere in the clinic.	✓			
	17. Cross-platform use.	✓			
	18. Prevents accidental misuse.		✓		
	19. Separate controls from display.				✓
	20. Separate stakeholder inputs.				✓
	21. Enjoyable user experience.	✓			
	22. Display username and role.			✓	

R – requirement, C – constraint, F – function, and M – means

### B. The Mass Vaccination Clinic Mobile Application

The developed app supports clinic staff manage patient numbers and vaccine preparation while automatically determining the current state of the clinic concerning vaccine availability. The app also provides an expected end-of-day dose value based on the anticipated number of vials to open to augment decisions for minimizing waste.

The interface enables staff to view and edit information at authorized clinics. Users can ‘enter’ each clinic on the app to view and edit the same information that other staff are viewing, which updates in real-time. Upon entering a clinic profile, users see the dashboard screen where they can view the state of the clinic regarding patient intake (i.e., the total number of patients: appointments, add-ons, and no-shows), vaccine preparation (i.e., the total number of doses prepared: drawn doses, pooled residual doses, and wasted doses), and information related to vial reconstitution (i.e., total expected vials, total punctured vials, and the total vials left in storage). The total number of expected vials is automatically calculated based on the number of expected doses required but can be manually entered if desired. Figure S3 of the supplementary material includes the final design of the dashboard, displaying the number of vials remaining to be punctured, the number of doses remaining to prepare, and the expected number of extra doses that might be available at the end of the clinic based on the status of the clinic and the total expected patients.

### C. Rapid Iterative Testing and Evaluation

Twenty-four participants implemented the app in their clinic, and 22 completed the follow-up interview and SUS survey (Table 3). Participant backgrounds included Pharmacists (11), Registered Practical Nurses (7), Registered Nurses (2), and Non-Clinical Staff/Security (4). Four participants were also students. The median age was 30 years, ranging from 19 to 61 years old. Participants reported working an average of 20.8 hours/week.

**TABLE 3:** Participant Demographics (N = 24)

Characteristics Participants, n (%)		Patient Intake H(45)	Vaccine Prep 13 (55)
Age (years)	18-24	3(27)	1(8)
	25-34	6(55)	6(46)
	35-44	2(18)	5(38)
	45-54	0(0)	0(0)
	55-64	0(0)	1(8)
Gender	Female	9(82)	9(69)
	Male	2(18)	4(31)
Highest Education	Unknown	1(9)	0(0)
	Diploma	8(73)	1(8)
	Bachelors	1(9)	4(31)
	Masters	1(9)	1(8)
	Doctorate	0(0)	7(54)
Clinic #	Clinic 1	7(64)	7(54)
	Clinic 2	2(18)	3(23)
	Clinic 3	2(18)	2(15)
	Clinic 4	0(0)	0(0)
	Clinic 5	0(0)	0(0)
	Clinic 6	0(0)	1(8)
Avg Hours/Week	1-10	1(9)	7(54)
	11-20	2(18)	3 (23)
	21-30	3(27)	0(0)
	31-40	5(45)	3(23)

#### 1) Usability Testing

From initial discussions among the research team and feedback from Clinic Leads and Vaccine Leads at Clinic #1, the initial wireframes, given in Figure S1 of the supplementary material, were updated to include inputting opened vials instead of drawn doses, adding colors to differentiate between input types, along with changes to information on the dashboard. The updated design was used at the beginning of the rapid iterative usability testing, given in Figure S2 of the supplementary material. Seven of the 24 participants (Two Clinic Leads and five Vaccine Leads among Clinic #1, #2 and #3) completed the tasks on an iPad or iPhone. All participants achieved correct results.

Interface navigation changes that were iteratively improved first included changing the colors of the upper tabs to match mainstream design principles for indicating the current tab a user was on. Also, participants often navigated between 2–3 bottom tabs before finding their desired screen and checked the dashboard or history lists to confirm their input was recorded. Therefore, the number of bottom tabs was reduced to three and the input history list was added below the corresponding input buttons to provide immediate visual feedback (Figure S3).

In Figure S2c of the supplementary material, *Extra Vials, Extra Doses* and *Update Vials* often needed to be re-explained to Clinic Leads. *Extra Vials* and *Extra Doses* were ultimately removed from the dashboard after observing their rare use during implementation. The automated *Update Vials* value which was calculated based on the *Expected Vials* was ultimately integrated into *Expected Doses* to consolidate the information to one value on the dashboard, which could be overridden if desired.

The participants also commented on the influence of title names on the dashboard on their intuitive understanding of the corresponding value. Vaccine Leads often asked for further explanations about how *Available Doses* and *Expected Doses* were calculated and how their inputs influenced it. We decided to re-name *Doses Available* to *End-of-Day Extra Doses* to improve clarity. A long-press feature was also implemented on each dashboard title to reveal a pop-up description or formula (Figure S3e), supporting the user’s understanding for how their inputs influenced the value. There were no additional usability issues to address by the 7^th^ participant’s usability testing.

#### 2) Clinic Implementation & Observations

The app was used at Clinic #1 for 31/37 days once the ethics applications were approved and used until the clinic’s last day. At Clinic #2, the app was used for 15 total days. At Clinic #3, the app was used for eight days during half-day shifts and one full-day shift. Two participants from Clinic #3 completed the study after using the app. For Clinic #6, the app was used for both days of the large vaccination event by five individuals and two of the researchers. One user consented to use their data from Clinic #6.

After observing the use of the app at the clinics, we categorized this data into the following themes displayed in Table 4. Observation themes related to (1) the experience of using the app on shared decision-making processes between the Vaccine Lead and Clinic Lead, (2) how the Clinic Lead used the app until they no longer needed the cognitive support it provided once patient intake numbers were low enough, (3) characteristics on how the two types of users interacted with the app and contributed information at each clinic, and (4) interest in trying the app.

**TABLE 4:** Observations on Application Implementation

Theme	Observations
Impacting shared decision-making	• Prompting decisions by the Vaccine Lead about vaccine preparation rates with Clinic Lead’s updates on patient numbers. • Huddling around the app dashboard to show others the status of the clinic concerning patient intake and vaccine preparation. • Focused conversations about how many extra patients or doses would be required. • Reducing the frequency or length of discussions throughout the day.
Using until the math is easier	• Clinic Lead enters patient intake information until approximately < 10 expected patients are remaining, and the Vaccine Lead has prepared all expected doses. • Clinic Lead is not always ‘zeroing’ the dashboard to balance patient intake numbers with vaccine preparation.
Information-interaction characteristics	Vaccine Lead: • Adding opened vials after pulling a batch. • Adding pooled doses after checking a batch. • Adding wasted doses as they occurred. • Checking for patient updates when entering new vaccine preparation information. • Checking for total patient updates when nearing the end of vaccine preparation. • Only checking for patient updates and not inputting vaccine preparation information. Clinic Lead: • Entering add-ons and no-shows in batches or as they occurred one-by-one. • Entering add-ons and no-shows as a new total value by deleting the old value in the list. • Regularly monitoring the top value change after inputting new information.
Interest in the app	• Showing the app’s interface and functionality to others and explaining to them how they are using it at the clinic to support information tracking and automating calculations.

#### 3) Feedback from Interviews

During the interviews, participants were asked to describe how they used the app at their clinic, how it influenced their collaboration and workflow, usability, and areas for improvement. Feedback concerning specific usability issues was iteratively updated when possible. The interviews were transcribed verbatim using Microsoft Streams and anonymized by the researchers. A thematic approach was taken to organize emerging concepts: (1) *system usability*, (2) *information display considerations*, (3) *impacts on patient care capacity*, and (4) *development opportunities*.

##### a: System Usability

The participants commented on aspects of system usability concerning the effectiveness, efficiency, and satisfaction of integrating the app within their clinic. Overall, participants described many positive aspects about the usability of the app. The interview data with respect to system usability is summarized in Table 5.

**TABLE 5:** Interview Feedback on System Usability

Sub-Theme	Description/Examples
Effectiveness	• Reducing Clinic Lead stress in communicating timely information across the clinic. • Reducing Clinic Lead workload for calculating the expected end-of-day doses. • Enabling the Vaccine Lead to make quicker adjustments with regular patient intake updates. • Reducing end-of-day workload by increasing confidence in earlier vaccine preparation. • Learnability through a user-friendly interface. • Transparency with clinic workflow processes. • Usefulness of timed entries by which user.
Efficiency	• Focused communication on specific values when discussing changes in numbers. • Reducing physical workload to communicate. • Better anticipating end-of-day doses/vials with access to frequent updates on patient numbers. • Real-time communication without having co-located workstations. • Provides access to updated information wherever they are in the clinic.
Satisfaction	• Trusting the calculations. • Trusting that others input accurate data. • Catching mistakes in paper-based tracking because of real-time inputs on the app. • Making the work more enjoyable. • Increasing time capacity to perform other aspects of their role in the clinic, such as medical emergencies or staff/patient questions.

##### b: Information Display Considerations

One of the most useful values on the dashboard was *Total Clients,* including the breakdown between *Appointments, Add-Ons,* and *No-Shows*. The Clinic Leads at Clinic #1 also relied on the *Doses Available* value (Figure S2c) to make decisions with the Vaccine Lead to open more vials. The dashboard evolved iteratively after being integrated at Clinic #2 and Clinic #3 to include automatically updating *Expected Vials, Vials to Prepare*, and *Doses to Prepare*. One of the Vaccine Leads also appreciated knowing how many vials remained with *Vials in Fridge*. Clinic Leads also wanted an option to enter *Administered Doses* to automate the number of doses available, and then compare this to *Total Doses* at the end of the day to check they were equal.

Along with the values themselves, participants also expressed that there were times when the label terminology was unclear or nonstandard to their clinic, including *Residual, Cancel*, and especially the top value, *Doses Available*. This further validated the need for a long press feature on each title to display an explanation or formula. Participants also wanted to view trends from previous days. Therefore, a history screen was added (Figure S3h). Regularly and infrequently used information was moved to the top and bottom of the dashboard, respectively.

##### c: Impacts on Patient Care Capacity

The clinical implementation of the app changed workflow behaviors of Vaccine Leads and Clinic Leads, who better understood each other’s information needs, and developed new habits of checking the dashboard for updates while frequently entering data. Some participants minimized or stopped using paper-based forms that they had created, where Clinic Leads expressed that implementing the app provided them with more time for other clinical responsibilities, such as attending to patients feeling faint or taking a washroom break, confident that the app was calculating the correct number of end-of-day doses. This lessened cognitive demand and increased time capacity led them to encourage participation by other staff because they wanted to use it to augment their decision-making.

Some Clinic Leads also mentioned that because the app provided accurate information reliably, this was a factor in their decision to stop physically counting doses near the end of the day, where they would otherwise halt patient intake. The app supported their confidence to continue patient flow in a safe manner, thereby avoiding patient crowding inside the clinic which is especially important during an airborne pandemic. Also, having greater confidence in the number of expected extra doses further augmented Clinic Leads’ decisions to provide extra caregivers, volunteers, and community members with the opportunity to be vaccinated against COVID-19 within the clinic’s operating hours.

In clinics where Clinic Leads primarily held decision-making responsibilities for patient intake and instructed vaccine preparation, Vaccine Leads felt less stressed when they noticed Clinic Leads were less stressed while using the app. In clinics where Vaccine Leads held primary decision-making responsibilities for vaccine preparation, they also felt less stress about preparing a surplus of vaccines because of the real-time patient intake updates, which may have reduced the risk of human error in their task of vigilantly checking each prepared syringe, ensuring that each patient received a non-expired COVID-19 vaccine at the correct dose. For example, a Vaccine Lead expressed a strong desire for all clinic staff to use the app because it caught a mistake on their daily Public Health report.

##### d: Development Opportunities

With Expo Go and Firebase, there was a challenge with the database connection timing out unexpectedly. When the system timed out, users had to log out and log in again. Most participants said this was inconvenient if they were entering new information, but they also saw it as a potential security feature if there was feedback on the timeout. All participants wanted a more extended period of inactivity before the app automatically forced them to log out. Fortunately, the app now supports persistent logins.

Participants also commented on the lack of notifications. They suggested including notifications about changes to *Total Clients* or *Doses Prepared*, or for the app to prompt team huddles for deciding on end-of-day dose preparation needs. Participants also wanted to refresh the input history manually to know if they had the most recent information. While the app does not yet support notifications, the dashboard and history can be refreshed by swiping down.

For clinics providing more than one brand of vaccine, they were required to have more than one clinic profile (i.e., one for Pfizer and one for Moderna). Users pressed two icons and then swiped through their list of clinics to access the other dashboard. While this met participants’ needs, the number of steps to switch clinics could be reduced.

Finally, participants commented that the app could be interoperable with the online booking and vaccine administration portals to eliminate human input error. However, participants were sometimes concerned that the portals were inaccurate and that many last-minute no-shows would be too uncertain for any system to handle. These collective results led to the current app design (Figure S3).

#### 4) System Usability Score

Participants evaluated their experience using the app quantitatively (Table 6). The average SUS score from the 22 participants who completed the study was 87, falling within the highest quartile (85-100) for usability with an adjective rating of ‘best imaginable’ [25]. We found no significant correlations between SUS scores and participant age, gender, education level, clinic role, or hours worked.

**TABLE 6:** System Usability Scale Response Statistics (N = 22)

Item	Statement	M	SD
1	I think that I would use this App frequently at the clinic.	4.77	0.43
2	I found the App unnecessarily complex.	1.55	0.60
3	I thought the App was easy to use at the clinic.	4.55	0.51
4	I think that I would need the support of a technical person to be able to use this App at the clinic.	1.55	0.74
5	I found the various functions in this App were well integrated.	4.27	0.46
6	I thought there was too much inconsistency in this App.	1.59	0.59
7	I would imagine that most people would learn to use this App very quickly.	4.55	0.60
8	I found the App very cumbersome/awkward to use at the clinic.	1.50	0.60
9	I felt very confident using the App at the clinic.	4.50	0.80
10	I needed to learn a lot of things before I could get going with this App at the clinic.	1.64	0.58

The mean rating for SUS score for each item on a 5-point scale for 22 participants: 1-strongly disagree, 2-disagree, 3-neutral, 4-agree, 5-strongly agree; mean (M); standard deviation (SD).

## IV. Discussion

The objectives of this study were two-fold: to understand (1) the design requirements for a mobile app that can support mass vaccination clinic stakeholders with their tasks on the ground floor, and (2) the usability of the app by primary stakeholders through implementation within these ad-hoc clinical environments. Ultimately, we achieved a final design through rapid iteration to meet the information needs of vaccine clinic stakeholders. By capturing the context of workflow processes across clinics to inform system design, and an iterative multi-disciplinary approach to improve usability, the implemented app was observed to augment decision-making and workflow processes for managing vaccine preparation with patient intake. Participants reported the app reduced stress and improved confidence in the context of having shared access to timely information and reliable, automated calculations, which gave them more time to provide patient care, maintain patient safety, and effectively meet vaccination needs.

### A. Designing for Changing Complexities

Despite the successes of implementing the app at the time of this study the COVID-19 vaccine rollout has evolved. Throughout the pandemic, vaccination clinics have experienced changes in vaccine preparation policy and regulations (e.g., storage requirements, and being able to pool doses) [27], and a rise and fall in community uptake combined with vaccine scarcity. Eligibility rules gradually changed, and additional vaccines were approved in different vial sizes. Inconsistencies between brands for mixing requirements, vaccine storage, expiry policies, optimizing the number of expected doses per multi-dose vial, and inventory issues also contributed to the complexity of this work domain [3], [28], [29], which continues to change.

The introduction of booster doses is a relatively recent complexity with vaccine brands that use half of the original dose for the booster dose. The dosing regimen for these brands brings complexity to the number of doses per vial, where a 14-dose vial could become a 28-dose vial. However, there may be restrictions on how many times a vial can be punctured (e.g., 20 times), requiring the Vaccine Lead to optimize the number of half and full doses to prepare. For example, a 14-dose vial with a maximum of 20 punctures could be optimized by drawing 12 half doses and eight full doses. While our analysis does not capture this recent complexity, it is important to recognize the challenge of developing flexible and adaptable systems for healthcare.

Given recent vaccine preparation changes, tracking vials that combine full and half doses may increase the complexity of using the app. Currently, users will require additional clinic profiles (i.e., a half dose profile and a full dose profile). However, Vaccine Leads may want to further optimize their vaccine preparation to handle this complexity and prevent waste, requiring an increased reliance on the Clinic Lead to provide updates on how many patients are receiving each brand of vaccine and at which dose size. Separated clinic profiles may reduce tracking errors and preparing a surplus of unneeded vaccine, as users would need to be cognitively aware of the clinic profile where they enter information. However, there may be other features that can support vaccine preparation with multi-brand and multi-dose vials, such as aggregating information across multiple clinic profiles. Additional inquiry is necessary to understand the evolving immunization campaign.

### B. Technology Adoption in Healthcare

Some of the primary factors influencing mobile technology adoption in healthcare include social and organizational factors that improve collaboration and coordination among healthcare professionals, workflow, efficiency, and workload [30]. While our study supports these factors in adoption, our participants also reported reduced stress from using the app. Despite being a system that depends on continuous human interaction to be useful for stakeholders’ information sharing, the implementation of the app was not perceived as a time burden, nor did participants express that it induced stress. Participants developed trust in the app, found it quick to learn how to use, felt more confident in their role, and were not concerned with privacy—factors which may have influenced adoption as similarly reported for other healthcare technologies [23], [31], [32], [33]. However, while users may trust the app, it is important to recognize that they must also trust each other to input accurate data.

Additionally, Clinic Leads who had clinical responsibilities may have been provided with greater bandwidth for supporting patient care emergencies or other disruptions to patient flow when using the app. By automating calculations to reduce errors and augmenting decisions to open more vials or find more patients, the app supported a demanding cognitive task in mass vaccination clinics. Further reducing the physical burden to share information also improved communication efficiencies which may have reduced the stress of Clinic Leads, thereby increasing their time capacity to support the patient care experience in a vaccination clinic. However, perceived liability risks, anticipation toward unknown benefits of the app, potential costs to learn and implement the app, and already present workplace stress may remain as barriers to new technology use in dynamic healthcare contexts [32].

### C. Strengths & Limitations

This study highlights the richness that a multi-disciplinary team of researchers and participants can bring to understanding the design and implementation of a mobile app for mass vaccination clinics during the COVID-19 pandemic. The developed app may reduce the stress of frontline workers by providing an efficient method of communication, greater transparency of vaccine and patient tracking information, and support for shared decision-making with respect to end-of-day doses across teams. This study further highlights the need for technology supporting cognitive work and meeting information sharing needs, with potential applications beyond COVID-19 vaccination.

The successful implementation of the developed app requires adoption by stakeholders. The impact of implementation was prominent in clinics with greater participation by Clinic Leads and Vaccine Leads who were excited to contribute to improving the app’s design and functionality. While participants in clinics with lower participation experienced benefits to their role while using the app, their experience may not have been fully realized without widespread adoption across most clinic staff. Greater effort by the research team to increase participant engagement, such as hosting a webinar, may have improved perceived excitement toward using the app.

Despite being developed to support the COVID-19 pandemic, participation in this study may have been impacted by the demanding nature of the pandemic. Potential participants were currently leading the rollout of the COVID-19 vaccines in clinics and handling the stress of daily operations. Interest in participating may have been impacted within clinics that had already designed other tools with the potential cost and pressure to implement a new system. However, the development of the app was justified, especially in clinics which saw most of the staff adopt the app, where participants felt that it significantly improved their experience coordinating and sharing information compared to other tools and artifacts, giving them more time to support the patient experience.

Finally, this study uses multiple methods to subjectively evaluate the implementation of the developed app through interviews and surveys. The objective implementation measure was the number of days the app was used at each clinic. However, the app has not been evaluated objectively with respect to workflow, stress, or decision-making.

### D. Future Directions

The mass vaccination clinic app continues to be refined from the results of this study and as the COVID-19 pandemic evolves. We are also looking into developing a web-app for greater accessibility across internet-enabled devices and easier deployment to potential users. Future research may involve implementing the app within other immunization settings or pharmacies to improve the app’s design and workflow integration, understand the factors influencing adoption, and capture the impact on patients’ care and immunization experiences, using audit logging to examine the intuitiveness of the app without being initially trained by a researcher. Also, while this study was limited to vaccine clinics in Ontario, Canada, the proliferation of mobile devices in low- middle-income countries may direct future global research, where COVID-19 vaccine equity continues to be an access issue [34]. Future clinical implementations may beneficially support immunization campaigns in these countries, further exploring contextual differences impacting adoption, usability, and cognitive workload, and the app’s potential impact on providing clinicians with a greater clinical capacity.

## V. Conclusion

We rapidly developed a mass vaccination clinic mobile app through contextual inquiry and design to support the cognitive demands associated with end-of-day doses decision-making. Through the RITE process and clinical implementation testing, participants experienced improved communication and workflow coordination combined with reduced stress about vaccine wastage and more time for clinical responsibilities. By providing clinic staff with the information needed for decision-making through mobile apps, there is significant potential to reduce their cognitive workloads and stress, which are inherently critical factors to improve the patient experience in mass vaccination clinics.

The rapid organization and preparation of vaccination clinics in non-healthcare environments provided a unique opportunity for developing partnerships with experts outside the traditional healthcare delivery field. This collaboration also brought challenges for rapidly building an app that can comprehensively meet the information needs of frontline workers across unique vaccination clinic settings. Given the successes of the app in this study, there is a need to further understand its impacts on frontline workers and patient care through clinical implementation in other healthcare contexts. With the global use of mobile devices, especially in low-resource settings, further understanding the mechanisms influencing the adoption of the app may provide valuable insight toward the integration of decision-support mobile apps in healthcare more broadly.
